# Increasing age, neural invasion, extramural vascular invasion, and short-course radiotherapy in locally advanced rectal cancer are associated with decreased tumor regression: a retrospective cohort study

**DOI:** 10.1007/s10151-025-03180-w

**Published:** 2025-07-27

**Authors:** O. F. Johnsen, R. Riis, S. Meltzer, K. M. Augestad

**Affiliations:** 1 Department of Gastrointestinal Surgery, Akershus University Hospital, Postboks 1000, 1478 Lørenskog, Norway; 2 Division of Surgery Campus Ahus, University of Oslo, Oslo, Norway; 3 Department of Oncology, Akershus University Hospital, Lørenskog, Norway

**Keywords:** Neoadjuvant therapy, Pathologic complete response, Chemoradiotherapy, Rectal neoplasms, Tumor regression grade

## Abstract

**Background:**

We investigated factors associated with pathologic complete response (pCR) and tumor regression grade (TRG) on the basis of clinical and pathological variables and their impact on cancer-free survival (CFS) after surgery for locally advanced rectal cancer (LARC).

**Methods:**

All patients with LARC undergoing neoadjuvant treatment before curative total mesorectal excision surgery were included in a prospective institutional database connected to the National Mortality Registry. One-way analysis of variance and Pearson’s chi-squared test were utilized to compare TRG groups. The Kaplan–Meier method and regression models were used to evaluate CFS, radiation modality, and staging factors.

**Results:**

Of 700 patients operated on for rectal cancer between 2014 and 2024, 159 (22.7%) had LARC without known systemic cancer. Twenty-seven patients had pCR (TRG 0, 17.0%), 46 TRG 1 (29.0%), 70 TRG 2 (44.0%), and 16 TRG 3 (10%). Poor tumor regression was associated with increasing age (*p* = 0.009), vascular (*p* < 0.001) and neural invasion (*p* = 0.005), less differentiated tumors (*p* < 0.001), short-course 5 Gy × 5 (*p* < 0.001) rather than long-course 2 Gy × 25 radiotherapy, and omission of neoadjuvant chemotherapy (*p* < 0.001). Older age was a predictor of short-course radiotherapy and omission of chemotherapy (*p* < 0.001). Follow-up time was 46.6 months (IQR 20–80.3 months). No differences were found in CFS between TRG groups 0–3 (*p* = 0.18), however pCR was associated with improved CFS (*p* = 0.047).

**Conclusions:**

Decreased tumor regression was associated with reduced radiotherapy and chemotherapy, neural and vascular invasion, poor differentiation, and increasing age. The latter may reflect reduced application of neoadjuvant treatment in older patients. Complete responders experienced increased cancer-free survival.

## Introduction

Colorectal cancer is the third most common cancer worldwide and the second most deadly. Rectal cancer accounts for about 40% of the total incidence of colorectal cancer [1]. Locally advanced rectal cancer (LARC) is treated with neoadjuvant radiation and often chemotherapy. Pathologists grade the tumor response to neoadjuvant treatment using different grading systems. The most favorable response is when no cancer cells are left in the tumor area, referred to as pathologic complete response (pCR) [2]. pCR has been reported for about 15–20% of patients who receive neoadjuvant treatment for rectal cancer [3, 4]. There is considerable variance in the effectiveness of neoadjuvant treatment on tumor regression, and evidence that favorable tumor response to neoadjuvant therapy is a good prognostic sign [3, 4]. There are limits in our knowledge of how to predict tumor regression for rectal cancer after neoadjuvant treatment. Most studies have focused on factors associated with favorable responses to neoadjuvant therapy. A few have also investigated factors associated with poor response to neoadjuvant treatment [5–9].

Understanding more about what causes varying responses to neoadjuvant therapy for rectal cancer will provide us with better tools to optimize oncologic treatment for patients with rectal cancer. This knowledge is essential in both a neoadjuvant setting and a watch-and-wait approach. This study aims to explore factors associated with tumor regression grade and investigate the association between tumor regression grade and cancer-free survival (CFS).

## Method

Patients who underwent surgery for rectal cancer at Akershus University Hospital from January 2014 to August 2024 were included in our prospectively collected institutional database. Data were obtained from this database and the Electronic Health Record System and linked to the National Mortality Registry. The Regional Committee for Medical and Health Research Ethics (REK 650256) approved the study.

### Patients—inclusion and exclusion criteria

Rectal cancer was defined as any adenocarcinoma located between the dentate line and 15 cm orally, measured on a rigid scope. On the basis of the Norwegian guidelines for colorectal cancer and decisions in multidisciplinary meetings, a proportion of these patients were treated with neoadjuvant therapy [10]. These patients formed our study group. Patients who were not operated on with curative intent or had known systemic metastasis before or at the time of rectal surgery were excluded from the analysis. The assessment concerning metastasis at the time of surgery was the basis of inclusion or exclusion. The most advanced rectal cancers (T4b) are routinely referred to our national cancer hospital Det Norske Radiumhospital (DNR) for rectal surgery. DNR is a specialized cancer center. Patients who needed beyond total mesorectal excision (TME) and patients who needed surgery for local recurrence were also operated on at DNR. Patients who had their rectal cancer operation at DNR were not included in the database or the analysis. Our national guidelines only recommend curative oncologic therapy without surgery in a study setting. All the patients in the dataset underwent rectal surgery [10].

### Indications for neoadjuvant treatment

The modality for clinical staging in the pelvis was magnetic resonance imaging (MRI), while computed tomography (CT) was the modality used to screen all patients with rectal cancer for systemic metastasis. On the basis of the preoperative MRI, the main indication for neoadjuvant radiation was a short distance from the tumor to the expected circular resection margin (CRM ≤ 2 mm). The guidelines also consider advanced clinical T stage, positive clinical N stage, and clinical suspicion of extramural vascular invasion (mrEMVI positive) as indications for neoadjuvant treatment.

For high tumors that have only invaded or grown into the peritoneum (T4a) and can be surgically removed with clear margins, either immediate surgery, short-course radiotherapy (5 Gy × 5), or chemoradiotherapy before surgery are options. If there is extensive infiltration into the peritoneal reflection and/or MRI signs suggestive of localized peritoneal carcinomatosis, where primary surgery is unlikely to achieve clear margins, preoperative chemoradiotherapy should be considered.

In the lower rectum, where there is little or no mesorectal fat and a short distance to structures such as the levator ani, puborectalis, prostate/seminal vesicles, or vagina/cervix, it is often unclear what the best approach is to ensure sufficient clear margins. Low-lying tumors carry a higher risk of recurrence, partly due to short margins to the circumferential resection margin (CRM) or due to intraoperative tumor perforation and positive resection margins (R1 resection), compared with higher tumors. Preoperative radiotherapy is indicated for most low rectal tumors (T2/advanced T2 to T4b), particularly when there is insufficient mesorectal fat between the tumor or tumor-involved muscularis and the mesorectal fascia (MRF) [10].

All patients included in the analysis received either a short-course of 5 Gy × 5 or a long-course of 2 Gy × 25 radiation therapy. Most patients also received neoadjuvant chemotherapy. Long-course radiotherapy was administered alongside 5-fluorouracil (5-FU)-based concomitant therapy, along with nonconcomitant neoadjuvant chemotherapy either before or after radiation (FOLFOX/(FOLFIRINOX)/CAPOX). This aligns with the total neoadjuvant treatment (TNT) definition in National Comprehensive Cancer Network (NCCN) Clinical Practice Guidelines in Oncology [11]. Short-course radiation was provided without chemotherapy or in combination with chemotherapy in a RAPIDO regime (*n* = 20) [10].

### Surgery and follow-up

All patients underwent total mesorectal excision approximately 10 weeks after neoadjuvant treatment was completed. Postoperative follow-up was performed according to Norwegian guidelines, focusing on detecting recurrent cancer [10]. The follow-up included computed tomography, clinical controls, and follow-up colonoscopy 5 years after surgery.

### Definition of tumor regression grade (TRG 0–3)

**Fig. 1:**
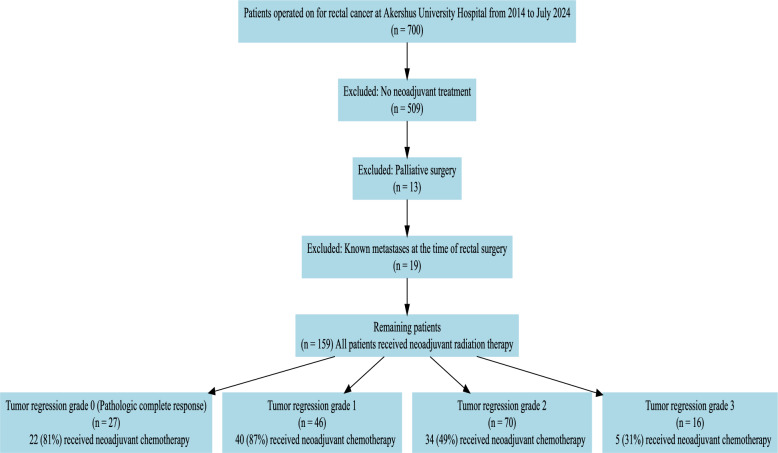
Study flow Consolidated Standards of Reporting Trails (CONSORT) diagram. All patients underwent rectal cancer surgery

We employed the Union for International Cancer Control (UICC)/American Joint Committee on Cancer (AJCC) TNM classification [12]. Pathologists evaluated all specimens after TME surgery and classified them according to a three-tiered tumor regression grading (TRG) system following Ryan [2]. The pathologist also classified the tumors according to pCR. Pathologic complete response was defined as the absence of visible cancer cells in the surgical specimen, including in the lymph nodes. Therefore, it was possible to convert Ryan’s three-tiered scoring system to a four-tier modified Ryan score [13]. TRG 0 corresponds to no viable cancer cells and is equivalent to a pCR. TRG 1 corresponds to only small groups of cancer cells, < 5% of the tumor area. TRG 2 is where residual cancer is outgrown by fibrosis, and there are cancer cells in 5–50% of the tumor. TRG 3 is where fibrosis is outgrown by cancer, which is defined as cancer cells in > 50% of the tumor area [2, 10] (Fig. 1).

### Statistics

Patients were categorized into four groups on the basis of TRG status. Numerical variables are presented as means with standard deviations. One-way analysis of variance (ANOVA) was employed to compare means across the different groups for the numerical variables. Categorical variables are presented as numbers and percentages. Pearson’s chi-squared test was used to compare categorical variables across groups.

The date of surgery, any local or systemic recurrence, and the date of death, if applicable, were recorded for all patients included in the analysis. Whether alive or deceased, the patient’s status is automatically updated in our electronic health record system through direct integration with the National Registry of Mortality. We conducted a patient status review for all patients included in the analysis during the first two weeks of December 2024 to determine their status as dead or alive by 1 December 2024. This provided data to calculate cancer-free survival, which was censored on 1 December 2024. Cancer-free survival, defined from the surgery date, was analyzed using the Kaplan–Meier method and evaluated with a log-rank test.

We used a binary logistic regression model to examine the association between short-course versus long-course radiation therapy with the predictor variables of age, mrMRF/sphincter involvement, mrEMVI, mrT stage, and mrN stage. The model was fit using the glm() function in R with a binomial distribution and logit link. Results are reported as odds ratio (OR) with 95% confidence interval (CIs), and significance was set at *p* < 0.05. The reference category for the tumor stage was mrT2, and the reference category for the nodal stage was mrN0. There were no observations for the mrT1 category in the dataset, so it was excluded from the analysis.

The relationship between chemotherapy and the predictor variables was analyzed using a similar logistic regression model.

We utilized a Cox regression model to evaluate the association between cancer-free survival, modality of radiation (short course versus long -course), age, and preoperative clinical (radiological) stage (cStage). Results are reported as hazard ratio (HR) with 95% confidence interval (CI), and significance was set at *p* < 0.05.

We conducted the statistical analysis using R version 4.4.1 in RStudio version 2024.09.0 build 375. The R packages used were broom, dplyr, ggplot2, gt, gtsummary, survival, survminer, and tidyverse (Tables 1, 2, 3).

**Table 1:** Comparison of TRG groups by clinical and demographic parameters

	Overall *N* = 159^1^	Complete response *N* = 27^1^	TRG 1 *N* = 46^1^	TRG 2 *N* = 70^1^	TRG 3 *N* = 16^1^	*p*-Value^2^
Sex						0.4
Female	62 (39%)	12 (44%)	14 (30%)	31 (44%)	5 (31%)	
Male	97 (61%)	15 (56%)	32 (70%)	39 (56%)	11 (69%)	
Age (years)	66 (11)	64 (10)	63 (10)	66 (12)	72 (9)	0.009
BMI (kg/m^2^)	26.4 (4.6)	27.0 (4.6)	25.9 (4.7)	26.4 (4.7)	26.0 (4.1)	0.8
ASA score						0.072
1	10 (6.3%)	1 (3.7%)	5 (11%)	4 (5.7%)	0 (0%)	
2	113 (72%)	22 (81%)	35 (78%)	47 (67%)	9 (56%)	
3	35 (22%)	4 (15%)	5 (11%)	19 (27%)	7 (44%)	
4	0 (0%)	0 (0%)	0 (0%)	0 (0%)	0 (0%)	
Unknown	1	0	1	0	0	
CEA level	7 (15)	6 (13)	7 (13)	7 (17)	8 (12)	> 0.9

^1^*n* (%); mean (SD)

^2^Pearson’s chi-squared test; one-way analysis of means not assuming equal variances

**Table 2:** Comparison of TRG groups by preoperative clinical staging parameters

	Overall *N* = 159^1^	Complete response *N* = 27^1^	TRG 1 *N* = 46^1^	TRG 2 *N* = 70^1^	TRG 3 *N* = 16^1^	*p*-Value^2^
mrT						0.084
1	0 (0%)	0 (0%)	0 (0%)	0 (0%)	0 (0%)	
2	14 (8.8%)	2 (7.4%)	2 (4.3%)	10 (14%)	0 (0%)	
3	97 (61%)	17 (63%)	24 (52%)	46 (66%)	10 (63%)	
4	48 (30%)	8 (30%)	20 (43%)	14 (20%)	6 (38%)	
mrN						0.14
0	73 (46%)	16 (59%)	17 (37%)	33 (47%)	7 (44%)	
1	53 (33%)	7 (26%)	13 (28%)	27 (39%)	6 (38%)	
2	33 (21%)	4 (15%)	16 (35%)	10 (14%)	3 (19%)	
Clinical stage						0.3
1	10 (6.3%)	2 (7.4%)	1 (2.2%)	7 (10%)	0 (0%)	
2	62 (39%)	14 (52%)	16 (35%)	25 (36%)	7 (44%)	
3	87 (55%)	11 (41%)	29 (63%)	38 (54%)	9 (56%)	
mrEMVI	44 (28%)	8 (30%)	16 (35%)	16 (23%)	4 (25%)	0.6

^1^*n* (%); mean (SD)

^2^Pearson’s chi-squared test; one-way analysis of means not assuming equal variances

**Table 3:** Comparison of TRG groups by preoperative clinical staging parameters

	Overall *N* = 159^1^	Complete response *N* = 27^1^	TRG 1 *N* = 46^1^	TRG 2 *N* = 70^1^	TRG 3 *N* = 16^1^	*p*-Value^2^
mrMRF/sphincter						0.3
Involving MRF	126 (80%)	18 (67%)	38 (86%)	55 (79%)	15 (94%)	
Involving sphincter	21 (13%)	7 (26%)	3 (6.8%)	10 (14%)	1 (6.3%)	
Not involving MRF/sphincter	10 (6.4%)	2 (7.4%)	3 (6.8%)	5 (7.1%)	0 (0%)	
Unknown	2	0	2	0	0	
mrAnal verge distance (cm)	6.3 (3.3)	6.3 (3.5)	6.8 (3.4)	5.8 (3.1)	6.8 (3.2)	0.5
mrPubo-rectal distance (cm)	3.01 (3.10)	3.03 (3.43)	3.58 (3.24)	2.64 (2.93)	2.97 (2.92)	0.5
mrTumor center (cm)	7.2 (3.3)	7.3 (3.5)	6.8 (3.3)	7.3 (3.2)	7.7 (3.8)	0.8
mrTumor diameter (cm)	4.79 (1.60)	4.66 (1.22)	4.84 (1.47)	4.69 (1.62)	5.28 (2.29)	0.7

^1^*n* (%); mean (SD)

^2^Pearson’s chi-squared test; one-way analysis of means not assuming equal variances

## Results

The median follow-up time in the TRG groups combined was 46.6 months (interquartile range 22.0–80.3). There was a statistically significant difference in age between the TRG groups. The patients in TRG group 3 were somewhat older. There was also a statistically significant difference between TRG groups and the use of short-course radiotherapy, where short-course therapy was associated with worse TRG. There was also a lower likelihood of chemotherapy use in TRG groups 2 and 3. There was also a statistically significant difference between the TRG groups regarding ypEMVI and ypNeural invasion, where the risk of these unfavorable characteristics increased with increasing TRG score. Regarding differentiation, there was a higher probability of the cancer being poorly differentiated and mucinous for the TRG 2 and TRG 3 groups. There were only six patients with mucinous tumors in the dataset. Most patients with ypT0 had a pathologic complete response and fell into the TRG 0 group. However, four patients were found to be ypT0N1. This explains why four patients with ypT0 are in the TRG 1 group. There was a statistically significant probability of a higher number of positive lymph nodes with higher TRG. There was also a shorter distance to the circular resection margin (CRM) in the TRG 3 group and a statistically significantly increased risk of a < 1 mm margin to CRM in the higher TRG groups. The pathologist measured the distance from the fibrotic noncancerous tumor tissue for the two patients with pathologic complete response who did not have an unknown CRM value (Tables 4, 5).

**Table 4:** Comparison of TRG groups by oncological and surgical treatment parameters

	Overall *N* = 159^1^	Complete response *N* = 27^1^	TRG 1 *N* = 46^1^	TRG 2 *N* = 70^1^	TRG 3 *N* = 16^1^	*p*-Value^2^
Neoadj. 5Gyx5	78 (49%)	11 (41%)	11 (24%)	41 (59%)	15 (94%)	< 0.001
Neoadj. 2Gyx25^3^	81 (51%)	16 (59%)	35 (76%)	29 (41%)	1 (6.3%)	< 0.001
Neoadj. chemo.^4^	101 (64%)	22 (81%)	40 (87%)	34 (49%)	5 (31%)	< 0.001
Laparoscopy	82 (52%)	13 (48%)	30 (65%)	32 (46%)	7 (44%)	0.2
Conv. to open	13 (8.2%)	1 (3.7%)	4 (8.7%)	6 (8.6%)	2 (13%)	0.8
Abdominal–perineal resection	75 (47%)	14 (52%)	19 (41%)	35 (50%)	7 (44%)	0.8
Hartmann’s opr.	14 (8.8%)	1 (3.7%)	3 (6.5%)	7 (10%)	3 (19%)	0.4
Anastomosis	68 (43%)	11 (41%)	24 (52%)	27 (39%)	6 (38%)	0.5
End stoma						0.4
No	70 (44%)	12 (44%)	25 (54%)	27 (39%)	6 (38%)	
End sigmoid	87 (55%)	14 (52%)	20 (43%)	43 (61%)	10 (63%)	
End ileo.	2 (1.3%)	1 (3.7%)	1 (2.2%)	0 (0%)	0 (0%)	
Anastomotic leakage, sinus and abscess	29 (18%)	5 (19%)	7 (15%)	11 (16%)	6 (38%)	0.2
Tumor/rectum perforation	14 (8.8%)	3 (11%)	1 (2.2%)	7 (10%)	3 (19%)	0.2
Adjuvant chemo.	5 (3.1%)	0 (0%)	1 (2.2%)	3 (4.3%)	1 (6.3%)	0.6

^1^ *n* (%)

^2^Pearson’s chi-squared test

^3^All these patients also received neoadjuvant chemotherapy

^4^All patients also received neoadjuvant radiation

**Table 5:** Comparison of TRG groups by histopathologic parameters

	Overall *N* = 159^1^	Complete response *N* = 27^1^	TRG 1 *N* = 46^1^	TRG 2 *N* = 70^1^	TRG 3 *N* = 16^1^	*p*-Value^2^
ypT						< 0.001
0	31 (19%)	27 (100%)	4 (8.7%)	0 (0%)	0 (0%)	
1	10 (6.3%)	0 (0%)	4 (8.7%)	5 (7.1%)	1 (6.3%)	
2	50 (31%)	0 (0%)	21 (46%)	26 (37%)	3 (19%)	
3	66 (42%)	0 (0%)	17 (37%)	39 (56%)	10 (63%)	
4	2 (1.3%)	0 (0%)	0 (0%)	0 (0%)	2 (13%)	
ypN						0.004
0	107 (67%)	27 (100%)	32 (70%)	39 (56%)	9 (56%)	
1	36 (23%)	0 (0%)	10 (22%)	22 (31%)	4 (25%)	
2	16 (10%)	0 (0%)	4 (8.7%)	9 (13%)	3 (19%)	
ypStage						< 0.001
0	27 (17%)	27 (100%)	0 (0%)	0 (0%)	0 (0%)	
1	47 (30%)	0 (0%)	22 (48%)	21 (30%)	4 (25%)	
2	33 (21%)	0 (0%)	10 (22%)	18 (26%)	5 (31%)	
3	52 (33%)	0 (0%)	14 (30%)	31 (44%)	7 (44%)	
4	0 (0%)	0 (0%)	0 (0%)	0 (0%)	0 (0%)	
ypEMVI	33 (21%)	0 (0%)	7 (15%)	18 (26%)	8 (50%)	< 0.001
ypNeural invasion	26 (16%)	0 (0%)	5 (11%)	15 (21%)	6 (38%)	0.005
Differentiation						< 0.001
Unclassifiable	62 (39%)	27 (100%)	18 (40%)	14 (20%)	3 (19%)	
Well-differentiated	38 (24%)	0 (0%)	14 (31%)	19 (27%)	5 (31%)	
Moderately differentiated	44 (28%)	0 (0%)	10 (22%)	30 (43%)	4 (25%)	
Poorly differentiated	8 (5.1%)	0 (0%)	3 (6.7%)	3 (4.3%)	2 (13%)	
Mucinous	6 (3.8%)	0 (0%)	0 (0%)	4 (5.7%)	2 (13%)	
Unknown	1	0	1	0	0	
CRM (mm)	7.0 (9.2)	4.5 (2.1)	9.0 (9.7)	6.9 (9.7)	3.0 (2.3)	0.042
Unknown	30	25	5	0	0	
CRM < 1 mm	20 (13%)	0 (0%)	4 (8,7%)	11 (16%)	5 (31%)	0.017
Quircke’s TME score						> 0.9
1	94 (62%)	16 (62%)	30 (68%)	40 (60%)	8 (53%)	
2	26 (17%)	5 (19%)	5 (11%)	13 (19%)	3 (20%)	
3	32 (21%)	5 (19%)	9 (20%)	14 (21%)	4 (27%)	
Unknown	7	1	2	3	1	
Distal resection margin (mm)	34 (17)	53 (15)	36 (19)	33 (15)	30 (19)	0.2
Unknown	32	24	8	0	0	
Number of lymph nodes	19 (9)	16 (10)	18 (8)	19 (9)	23 (9)	0.14
< 12 lymph nodes	30 (19%)	10 (37%)	8 (17%)	10 (14%)	2 (13%)	0.064
Number of pos. lymph nodes	0.93 (2.01)	0.00 (0.00)	0.72 (1.41)	1.16 (1.95)	2.13 (3.95)	0.004

^1^*n* (%); mean (SD)

^2^Pearson’s chi-squared test; one-way analysis of means not assuming equal variances; one-way analysis of means

**Fig. 2:**
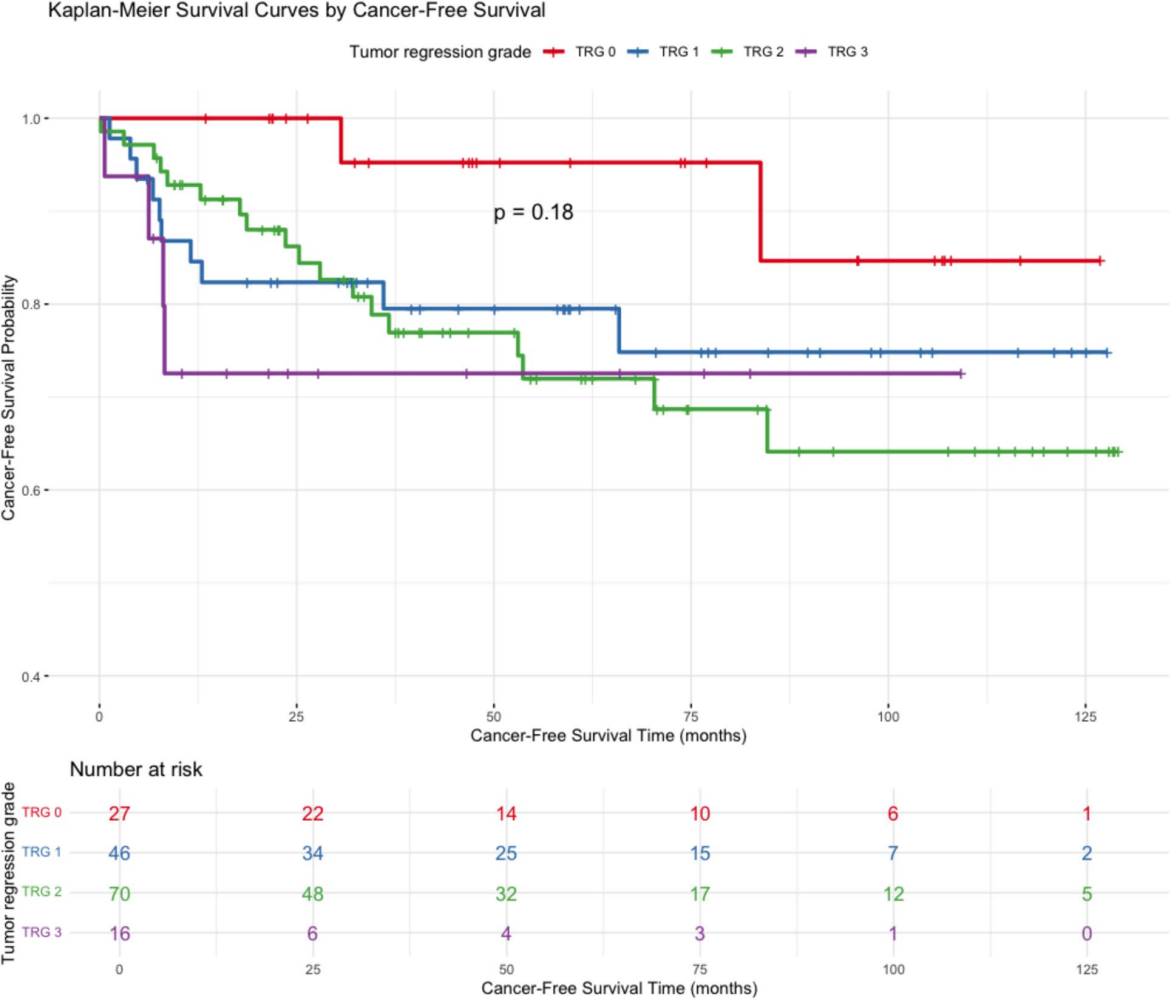
Kaplan–Meier cancer-free survival curves by TRG groups

**Fig. 3:**
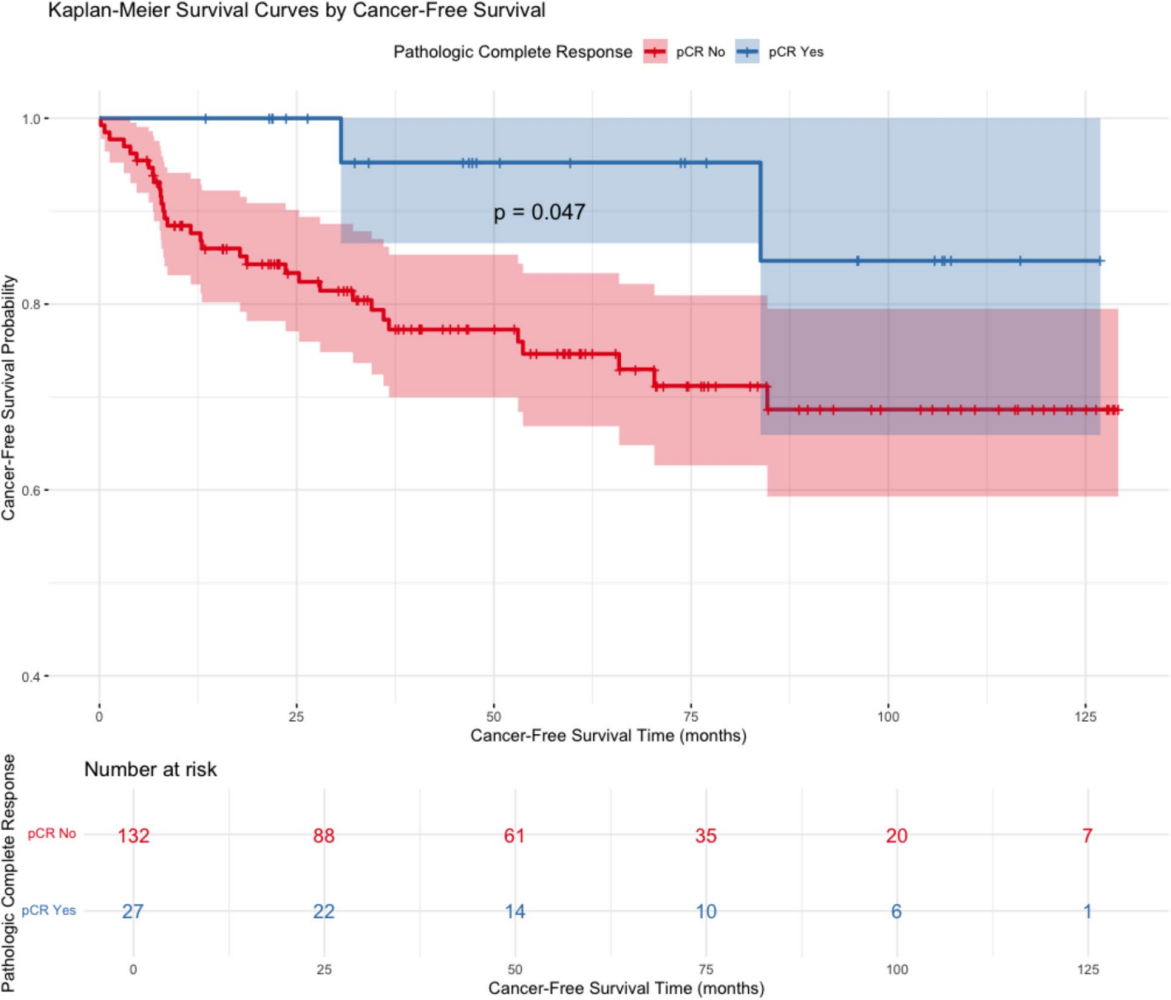
Kaplan–Meier cancer-free survival curves by pathological complete response versus non-pCR

CFS presented as Kaplan–Meier curves (Fig. 2) did not show a statistically significant difference between the four TRG groups (*p* = 0.18). However, there was a statistically significant difference in CFS when divided into two groups, one with pCR and the other without pCR (*p* = 0.047, Fig. 3). There were only two events, both non-cancer-related, in the group with pCR. We did not find an association between pretreatment carcinoembryonic antigen (CEA) or the tumor distance to the anal verge and TRG.

The binary logistic regression model predicting short-course radiotherapy versus long-course radiotherapy on the basis of the predictor variables (age, mrMRF/sphincter involvement, mrEMVI, mrT-stage, and mrN-stage) showed an association between higher age and higher odds ratio of use of short-course radiotherapy versus long-course radiotherapy. The model also showed an association between mrEMVI and short-course radiotherapy (Table 6). There were 78 patients in the short-course group and 81 in the long-course group. There was also an association between higher age and less use of chemotherapy when analyzed with the same type of regression model (Table 7); 101 patients received chemotherapy, while 58 did not.

**Table 6:** Odds ratio of the use of short-course (5 × 5 Gy) versus long-course (25 × 2 Gy) radiation therapy on the basis of age, MRF, EMVI, and T and N stage

	Estimate and SE			Confidence interval	
	Odds ratio	Standard error	*p*-Value	Lower	Upper
Per additional year of age	1.037	0.016	0.0262	1.005	1.071
mrMRF/sphincter involvement^1^	0.672	0.747	0.5948	0.133	2.761
mrEMVI^2^	2.474	0.419	0.0307	1.106	5.769
mrT3^3^	0.792	0.622	0.7078	0.224	2.673
mrT4^3^	0.373	0.679	0.1465	0.095	1.402
mrN1^4^	0.611	0.394	0.2111	0.280	1.320
mrN2^4^	0.451	0.480	0.0970	0.172	1.141

Reference: ^1^without mrMRF/sphincter involvement, ^2^without mrEMVI, ^3^mrT2, ^4^mrN0

**Table 7:** Odds ratio for the use of chemotherapy on the basis of age, MRF, EMVI, and T and N stage

	Estimate and SE		*p*-Value	Confidence interval	
	Odds ratio	Standard error		Lower	Upper
Per additional year of age	0.939	0.019	0.0008	0.904	0.973
mrMRF/sphincter involvement^1^	1.599	0.743	0.5276	0.365	7.194
mrEMVI^2^	2.167	0.465	0.0960	0.891	5.592
mrT3^3^	1.676	0.648	0.4255	0.476	6.252
mrT4^3^	4.590	0.724	0.0352	1.135	19.94
mrN1^4^	1.008	0.413	0.9853	0.446	2.272
mrN2^4^	1.949	0.541	0.2169	0.693	5.902

Reference: ^1^without mrMRF/sphincter involvement, ^2^without mrEMVI, ^3^mrT2, ^4^mrN0

The Cox regression model did not show a significantly increased hazard rate for death or cancer recurrence for those who received short-course radiotherapy versus those who received long-course radiotherapy. In addition, patient age or clinical stage was not significant in this regression model. There were only 34 events of recurrence or death in the follow-up period (Table 8).

**Table 8:** Cox regression model: hazard rate for death or cancer recurrence

	HR^1^	95% CI^2^	*p*-Value
5Gy × 5^3^	1.30	0.64, 2.66	0.5
Age	1.02	0.98, 1.05	0.3
cStage 2^4^	1.80	0.23, 14.2	0.6
cStage 3^4^	2.54	0.34, 19.2	0.4

^1^HR, hazard ratio

^2^CI, confidence interval

^3^Versus 2 Gy × 25

^4^Versus cStage 1

## Discussion

In 159 patients undergoing neoadjuvant treatment prior to TME, we analyzed the impact of tumor regression grade on significant outcomes such as cancer-free survival. We also evaluated predictors of TRG, including radiation and chemotherapy. The key finding is that advanced age, shorter radiation courses, and reduced chemotherapy use are significantly associated with poor tumor regression.

## Research in context

Age predicts short-course versus long-course radiotherapy when adjusted for mrMRF/sphincter involvement, mrEMVI, mrT, and mrN. Older patients were also less likely to receive chemotherapy. This may reflect that oncologists are more reluctant to subject older patients to more intense regimes of radiation and chemotherapy. However, our data do not include enough events to create robust regression models that could suggest important associations between CFS and the reduced intensity of neoadjuvant treatment for older patients.

The association between mrEMVI and short-course radiotherapy could be explained by the fact that the Norwegian guidelines for colorectal cancer recommend the RAPIDO regime (5 GY × 5 and chemotherapy) for patients who are mrEMVI positive [10]. Most previous studies indicate that older patients with rectal cancer receive less radiotherapy than younger patients [14–18]. It seems established that short-course versus long-course regimes have less toxicity [19]. However, whether older age increases the risk of radiation toxicity is controversial [16–18].

Some studies report a higher incidence of pathologic complete response after long-course versus short-course radiotherapy. Still, many of these studies are small, and the difference is often not statistically significant [20–23]. One study also reported similar pCR rates between short-course radiotherapy and two cycles of neoadjuvant chemotherapy versus long-course radiochemotherapy [24]. A meta-analysis reported an increased likelihood of pathologic complete response with long-course versus short-course radiotherapy. However, this increase in pCR did not translate to better overall survival or local recurrence outcomes [25]. Some studies suggest that delaying surgery after short-course radiotherapy improves the likelihood of pathological complete response compared with operating immediately after radiation (surgery after less than 1 week versus 4–8 weeks) [26]. This delayed surgery still occurred sooner after completing radiotherapy compared with the timing in our cohort. We found an association between the use of short-course radiotherapy and poor tumor regression (TRG 3). This was not found in an earlier study by Ng et al. [9]. To our knowledge, our study is the first to have found such an association. It must be mentioned that fewer TRG 3 patients received chemotherapy. This could influence the tumor regression grade.

Unlike earlier studies, we did not find a higher risk of not achieving pathologic complete response with higher clinical stages such as mrT4 or mrN2. We also did not find an association between pretreatment CEA levels and TRG [5, 6]. We did, however, following earlier studies, find an association between tumor regression grade and tumor differentiation [5, 6]. Some studies have reported an association between pCR and the tumor distance to the anal verge [8, 27]. We did not find such associations. Like in earlier studies, we discovered an unfavorable association between vascular invasion and TGR [7, 27].

A meta-analysis by Kasi et al. showed a higher proportion of pathological complete response (pCR) in the total neoadjuvant therapy (TNT) group compared with those who received neoadjuvant chemoradiation followed by adjuvant chemotherapy [28]. In our study, more TRG 0 and TRG 1 patients received neoadjuvant chemotherapy. Since all patients in our study also received neoadjuvant radiation—with or without concomitant chemotherapy—we consider these patients to have received TNT. Our findings align with those of Kasi et al. regarding pCR rates. We have relatively long-term follow-up data for patients treated with TNT, as defined in NCCN Clinical Practice Guidelines in Oncology and Kasi et al. [11, 28]. However, because only five patients who did not receive neoadjuvant chemotherapy went on to receive adjuvant chemotherapy, we lack a control group comparable to that in the referenced study [28]. We can therefore not make any meaningful comparison using our data between TNT patients and patients receiving adjuvant chemotherapy.

## Strengths and limitations

There are limitations: First, the data were analyzed retrospectively and were from a single center. We also did not have access to genetic information (*KRAS*, *BRAF*, and *MSI*), which may influence tumor responses to neoadjuvant treatment [29, 30].

Secondly, the association between TRG groups ypEMVI, ypNeural invasion, and the number of cancer-positive lymph nodes may significantly affect the risk of systemic metastasis. However, the events are too few in the dataset to make a meaningful separate analysis of local and systemic recurrence.

Thirdly, one should be cautious when evaluating the statistical significance of cancer-free survival and pCR status. As mentioned, patients were excluded from the analysis if systemic cancer disease was diagnosed before surgery. Some patients had inconclusive radiology findings regarding systemic metastases owing to small nodules. While some patients later proved to have distant metastasis, others did not. The date for metastasis was established as when the diagnosis was made. The log-rank test would not have yielded a significant result with one fewer event in the non-pCR group. Larger datasets do, however, consistently demonstrate an association between pathologic complete response and excellent long-term outcomes concerning local and distant recurrence [4]. Less favorable tumor regression in the higher TRG groups did not result in a statistically significant difference in cancer-free survival when comparing all groups. Notably, the TRG 3 group was very small.

Interpreting the regression model (Table 7) predicting less use of chemotherapy with increasing age requires caution, as the smallest group has only 58 patients, which may be insufficient given the seven predictor categories.

Our Cox regression model did not find a significant increase in hazard rate for death and cancer recurrence for short-course versus long-course radiotherapy, adjusted for age and preoperative clinical stage. This could be because the model is too small to capture significant confounders. With only 34 events, we are already at risk of oversaturating the model and cannot justify adding more variables. One should, therefore, be cautious in interpreting this model (Table 8).

A strength of the study is that we used reliable data from our prospectively collected institutional database linked to mortality data from the National Registry of Mortality. We also had a median follow-up of 47 months postoperatively, which makes this one of the most comprehensive studies assessing TRG as an independent predictor for cancer-free survival.

## Conclusions

Poor tumor regression was associated with older age, less radio- and chemotherapy, poorly differentiated and mucinous tumors, as well as pathological signs of vascular and neural invasion.

## Data Availability

The data used in this study come from an internal quality register and are protected under Norwegian law. Due to privacy regulations, the data cannot be publicly shared. Access may be granted upon request, subject to ethical approval and compliance with applicable laws.
